# Diversity of lactic acid bacteria in dadih produced by either back-slopping or spontaneous fermentation from two different regions of West Sumatra, Indonesia

**DOI:** 10.14202/vetworld.2019.823-829

**Published:** 2019-06-15

**Authors:** Chandra Utami Wirawati, Mirnawati Bachrum Sudarwanto, Denny Widaya Lukman, Ietje Wientarsih, Eko Agus Srihanto

**Affiliations:** 1 Graduate School of Veterinary Public Health, Bogor Agriculture University, Bogor, Indonesia; 2 Study Program of Food Technology Lampung State Polytechnic, Lampung, Indonesia; 3 Department of Animal Diseases and Veterinary Public Health, Faculty of Veterinary Medicine, Bogor Agricultural University, Bogor, Indonesia; 4 Department of Veterinary Clinic, Reproduction and Pathology, Faculty of Veterinary Medicine, Bogor Agricultural University, Bogor, Indonesia; 5 Lampung Veterinary Office, General Directorate Animal Husbandry and Healthiness, Agricultural Ministry Republic of Indonesia, Lampung, Indonesia

**Keywords:** back-slopping, dadih, lactic acid bacteria, spontaneous fermentation

## Abstract

**Aim:**

Dadih samples from two different origins (Kamang and Gadut in West Sumatra) manufactured with different methods (back-slopping or spontaneous fermentation) were evaluated for the diversity of lactic acid bacteria (LAB).

**Materials and Methods:**

Four dadih samples manufactured with two different fermentation methods were obtained from Kamang and Gadut regions. Both genotypic and phenotypic characteristic (16S rRNA partial gene sequence analysis and carbohydrate fermentation profile) were used to analyze the diversity of dadih LAB population.

**Results:**

This study showed that LAB count in back-slopping fermented dadih was one log cycle higher than spontaneous fermented dadih. LAB isolates from the two regions were divided into three genera, namely *Lactococcus*, *Lactobacillus*, and *Pediococcus*. Sequencing results showed that 41.6% (five isolates) were identified as *Lactococcus lactis* ssp. *lactis*, 25% (three isolates) were identified as *Lactobacillus plantarum* ssp. *plantarum*, 16.6% (two isolates) were identified as *L*. *lactis* ssp. *cremoris*, and 8.3% (one isolate each) were identified as *Pediococcus pentosaceus* and *Lactobacillus pentosus*.

**Conclusion:**

Five species were determined in back-slopping fermented dadih, i.e., *L. lactis* ssp. *lactis*, *L. lactis* ssp. *cremoris*, *L. plantarum* ssp. *plantarum*, *L. pentosus*, and *P. pentosaceus*. On the other hand, spontaneous fermented dadih only contained three different species, namely *L. lactis* ssp. *lactis*, *L. lactis* ssp. *cremoris*, and *L. plantarum* ssp. *plantarum*. This research showed that back-slopping fermentation offers greater abundance and diversity compared to spontaneous fermentation in dadih.

## Introduction

Dadih is naturally fermented buffalo milk from West Sumatra and has become an integral part of Minangkabau diet. The dadih has “yogurt-like” consistency, i.e. soft texture, whitish cream color, sour, and pleasant taste. Dadih surface is smooth and shiny, clean without air bubbles in the middle of the product [1]. The fermentation process occurs spontaneously; bamboo tubes are filled with fresh buffalo milk and each tube is covered with banana leaves that are then incubated at room temperature (28-30°C) for approximately 24-48 h [2]. Nevertheless, there are still some artisans that apply back-slopping fermentation methods during dadih production. Back-slopping method is carried out by adding a small amount of fermented products into fresh ingredient and then is left to be fermented in room temperature. Finally, a stable microbial community is formed after a few cycles [3].

Various types of indigenous microflora dominated by lactic acid bacteria (LAB) in spontaneous fermentation cannot be fully controlled or correctly predicted, even though this process has been practiced for many years [4]. Microorganism might be derived from milk or the environment, i.e. bamboo tubes, banana leaves, or artisans. This traditional processing of dadih is obtained from generation to generation and is accompanied by a low quality of sanitation and hygiene during the process. The biggest risks for natural milk fermentation are the low hygiene conditions during processing, the high initial contamination, and the absence of a heating stage before the process [5]. These problems can be overcome gradually by applying the back-slopping method so that the uniformity and safety of the dadih can be correctly predicted. Various types of LAB are isolated from dadih and play a role in spontaneous fermentation, specifically *Leuconostoc paramesenteroides*, *Lactobacillus casei* ssp*. rhamnosus*, *L*. *casei* ssp*. casei*, *Lactococcus lacti*s ssp. *lactis*, *Lactobacillus brevis*, *Lactobacillus plantarum*, *Lactobacillus paracasei*, *Lactobacillus fermentum*, *Pediococcus pentosaceus*, *L. rhamnosus*, and *Enterococcus faecium* [6-9].

Spontaneous and back-slopping fermentation methods are still performed during dadih production in Gadut and Kamang regions, West Sumatra. No study has been conducted on comparing these two fermentation methods that are applied during dadih production. We assume that LAB population is affected by the different fermentation methods. The aim of this study was, therefore, to highlight the diversity of LAB isolated from dadih in Gadut and Kamang, West Sumatra.

## Materials and Methods

### Ethical approval

No live animals were used in the present study. Samples were purchased from herdsmen from Kamang and Gadut region in West Sumatra.

### Study area

Kamang is located in Agam District; meanwhile, Gadut is located in Limapuluhkota District. Their distance from Bukittinggi city is 16 km and 42.4 km, respectively.

### Sampling

Four dadih samples were purchased from two regions in West Sumatra, namely Kamang and Gadut. Dadih sample from Kamang was processed with back-slopping fermentation by adding approximately 50 g (1 tablespoon) of previous dadih into 1600 ml of fresh milk and then placed into bamboo tube. Fermentation process occurred for 48 h at room temperature. Spontaneous fermentations were applied in dadih from Gadut. Fresh buffalo milk was inserted into the bamboo tube and let it fermented for 48 h at room temperature. pH of dadih sample was measured using Hanna HI8424 microcomputer pH meter and then 5 g were aseptically removed in a sterile tube and transferred to the laboratory under cool condition (4°C) for further analyses.

### Isolation

About 1 g of each dadih sample was mixed with 45 ml of sterile NaCl 0.85%. An appropriate dilution (10^7^-10^8^) was made and inoculated on de Man-Rogosa-Sharpe (MRS) Agar (Merck) + 0.5% CaCO_3_ medium by double layer technique and incubated at 37°C for 48 h. The number of LAB colonies was expressed as log unit colony count (log cfu) per gram sample. Ten colonies with the surrounding clear zone were randomly selected in each plate. Non-motile rod and coccus, Gram-positive and catalase-negative isolates were plated on fresh MRS plates to obtain a pure culture. LAB isolates were stored in 10% (v/v) of sterile glycerol at −20°C [10].

### Molecular identification

The genome DNA of the selected LAB isolates was extracted using Presto^™^ Mini gDNA Bacteria Kit (Geneaid). The DNA pellets were suspended in 50 µL TE buffer (Tris-EDTA) and stored at −20°C. LAB 16S rRNA gene was amplified using a universal primer 27F (5’-AGAGTTTGATCCTGGCTCAG-3’) and 1492R (5’-GGTTACCTTGTTACGACTT-3’) [11]. A total volume of polymerase chain reaction (PCR) of 50 ml consisted of 25 µL MyTaq HS Red Mix (Geneaid), 2 µl of each primer (10 pmol), and distilled water to achieve the final volume. Amplification was performed in Takara PCR Thermal Cycler SimpliAmp with PCR conditions as follows: Pre-heating at 94°C for 1.5 min, denaturation at 95°C for 30 s, annealing at 50°C for 30 s, and elongation at a 72°C for 1.5 min, this cycle was repeated at 30 cycles and finally performed at 72°C for 5 min. PCR products were electrophoresed in 1% agarose gel. PCR products were sent to the 1^st^ BASE for sequencing 16S rDNA gene. The sequencing results were subjected into National Center for Biotechnology Information (NCBI) GenBank (www.ncbi.nlm.nih.gov) and basic local alignment search tool (BLAST) was adopted to search GenBank database for sequence homology analysis to determine the genera of LAB. Species with similarity more than 97% was considered as the same. Phylogenetic tree of LAB was constructed with MEGA 10 software, (Proprietary Freeware, Pennsylvania State University), and neighbor-joining methods were performed to test confidence with bootstrap data set of 1000 times [12].

### Fermentation profile

Identification of LAB isolates was carried out through observation of carbohydrate fermentation patterns using API^Ò^ 50 CHL kit (bioMérieux, France). The fermentation profile of isolates was determined using APILAB Plus software version 3.3.3. from bioMérieux [12].

## Results

### Isolation

Fifty-three presumptive LAB colonies with clear zone around the colony were randomly selected. The morphological test results showed 31 isolates that were categorized as non-motile rod and coccus, Gram-positive bacteria, and negative to catalase (Table-1). Based on antibacterial and proteolytic activity (data not shown), 12 LAB isolates were selected for further analysis.

**Table-1:** Number of isolates, pH, total LAB, and antimicrobial properties against *Escherichia coli,* *Staphylococcus aureus*, *Salmonella* Typhi, and proteolytic activity.

Variables	Gadut	Kamang
Number of isolates	12	19
pH	4.57	4.52
Total LAB (log cfu/g)	8.56	9.04
Isolates with antimicrobial properties	9	13
Isolates with proteolytic activity	5	7

LAB=Lactic acid bacteria

### Molecular identification

**Figure-1:**
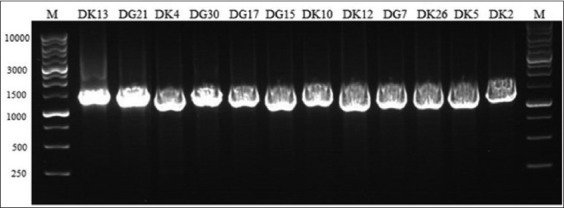
Polymerase chain reactions product lactic acid bacteria (LAB) isolates in 1% agarose gel. Line 1 and 14 (Ladder DNA 1 kb); Line 2-13 (LAB isolate); DK=Dadih from Kamang, DG=Dadih from Gadut.

Twelve isolates were determined and compared with the partial 16S rRNA gene sequence. The DNA size of all isolates was approximately 1000-1500 bp (Figure-1). To confirm the species, these sequences were determined and compared with related bacteria using BLAST program at NCBI. LAB isolates from the two regions were divided into three genera, namely *Lactococcus*, *Lactobacillus*, and *Pediococcus*. Sequencing results showed that 41.6% (5 isolates) were identified as *L. lactis* ssp. *lactis*, 25% (three isolates) were identified as *L. plantarum* ssp. *plantarum*, 16.6% (two isolates) were identified as *L*. *lactis* ssp. *cremoris*, and 8.3% (one isolate each) were identified as *P. pentosaceus* and *Lactobacillus pentosus* (Table-2).

**Table-2:** Identification of 12 LAB isolate by 16S rRNA sequence.

Isolate code	Origin	Species	% ID in NCBI	Accession number
DK13	Kamang	*Lactobacillus pentosus*	99	CP032757.1
DG21	Gadut	*Lactobacillus plantarum* ssp. *plantarum*	99	KY762263.1
DK4	Kamang	*Lactococcus lactis* ssp. *lactis*	97	KF879153.1
DG30	Gadut	*Lactococcus lactis* ssp. *cremoris*	98	MF098152.1
DG17	Gadut	*Lactobacillus plantarum* ssp. *plantarum*	98	CP031771.1
DG15	Gadut	*Lactococcus lactis* ssp. *cremoris*	99	MF098152.1
DK10	Kamang	*Pediococcus pentosaceus*	99	AB494722.1
DK12	Kamang	*Lactococcus lactis* ssp. *lactis*	98	KJ095659.1
DG7	Gadut	*Lactococcus lactis* ssp. *lactis*	98	KF148962.1
DK28	Kamang	*Lactococcus lactis* ssp. *lactis*	98	KF879153.1
DK5	Kamang	*Lactococcus lactis* ssp. *cremoris*	98	KM485587.1
DK2	Kamang	*Lactobacillus plantarum* ssp. *plantarum*	98	KY762263.1

DK=Dadih from Kamang, DG=Dadih from Gadut, LAB=Lactic acid bacteria, NCBI=National Center for Biotechnology Information

### Diversity and phylogenetic tree

**Figure-2:**
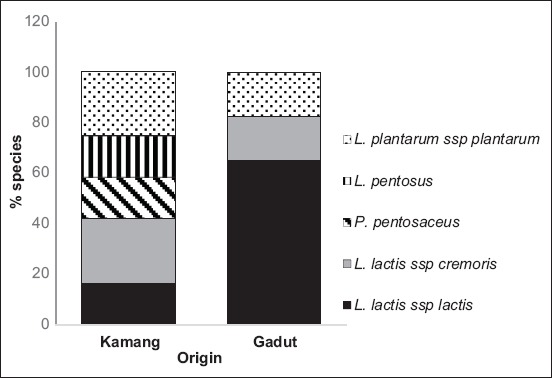
Greater diversity in lactic acid bacteria Dadih from Kamang origin than Gadut origin.

LAB in dadih from Kamang (DK) origin had greater diversity than that originated from Gadut (Figure-2). It consisted of three genera, namely *Lactococcus*, *Pediococcus*, and *Lactobacillus*, while that in dadih from Gadut was only dominated by two genera, i.e. *Lactococcus* and *Lactobacillus*.

**Figure-3:**
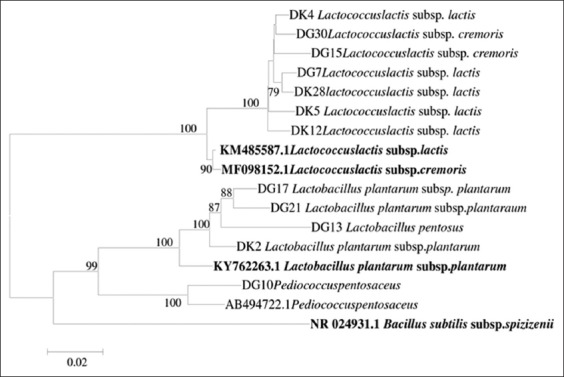
Neighbor-joining tree of LAB dadih from Kamang and Gadut origin showing phylogenetic relationship between two genera cocci and bacilli. Bacillus subtilis ssp spizizenii as an out group.

The neighbor-joining method and p-distance parameter model were applied to construct the phylogenetic tree among the aligned 16S rRNA sequences of all LAB isolates and reference strains (Figure-3). In the study, LAB isolates phylogenetic tree consisted of two clusters based on their genetic type. The first cluster belonged to *Lactococcus* genus, which was composed of one species, namely *L. lactis*. The second cluster belonged to *Lactobacillus* and *Pediococcus* genera. Lactobacillus genus consisted of two species, i.e. *L. plantarum* ssp. *plantarum* and *L. pentosus*, while *Pediococcus* genus was identified as *P. pentosaceus*.

### Fermentation profile

Fermentation profile of LAB isolates was carried out based on the ability to ferment various carbohydrates using API 50 CHL Kit test (Table-3). API 50 HCL kit test confirmed an accuracy rate above 99% apart from DK13 (96.8%) and DK10 (97%) isolates, although these results are still relatively high. The fermentation profile supports the results of genotypic identification based on 16S rRNA partial sequences gene and matches with their biochemical characteristics.

**Table-3:** Fermentation profile of LAB isolates from Kamang and Gadut in API 50 CHL kit test.

Isolate code*	Accuration level (%)	Identification
DK13	96.8 very good identification	*Lactobacillus pentosus*
DG21	99.9 excellent identification	*Lactobacillus plantarum* ssp. *plantarum*
DK4	99.8 very good identification	*Lactococcus lactis* ssp. *lactis*
DG30	99.9 excellent identification	*Lactococcus lactis* ssp. *cremoris*
DG17	99.9 excellent identification	*Lactobacillus plantarum* ssp. *plantarum*
DG15	99 very good identification	*Lactococcus lactis* ssp. *cremoris*
DK10	97 very good identification	*Pediococcus pentosaceus*
DK12	99.9 excellent identification	*Lactococcus lactis* ssp. *lactis*
DG7	99.9 excellent identification	*Lactococcus lactis* ssp. *lactis*
DK28	99.8 very good identification	*Lactococcus lactis* ssp. *lactis*
DK5	99.8 very good identification	*Lactococcus lactis* ssp. *cremoris*
DK2	99.9 excellent identification	*Lactobacillus plantarum* ssp. *plantarum*

* DK=Dadih from Kamang, DG=Dadih from Gadut, LAB=Lactic acid bacteria

## Discussion

Dadih fermentation methods in the West Sumatra are generally classified as spontaneous. The product quality and safety are associated with the diversity and the population of microorganisms contained in the buffalo milk. Quality limitations from spontaneous fermentation caused a shift to back-slopping fermentation, which has been proven to accelerate the fermentation rate and increase safety, sensory quality, and product nutrition content [13-15]. Dadih from Gadut region was produced by spontaneous fermentation. On the other hand, specifically in Kamang, dadih is processed by back-slopping fermentation. The difference between these methods could dramatically affect the diversity of LAB as a dominant bacterial agent for dadih fermentation.

The results of the study (Table-1) showed that pH of the product ranged between 4.52 and 4.57. LAB relative abundance in dadih fermented with back-slopping method was 1 log cycle higher than that produced by spontaneous fermentation. Similar results were also shown in Kivunde and Ogi product that are fermented foods made from cassava and corn [13,16]. This high bacterial population was possibly an effect of back-slopping method application. A small amount of product from previous fermentation that was added in the buffalo milk not only caused this increase but also accelerated the fermentation time.

The results of the genotype analysis of the 12 LAB isolates showed that they were grouped into three genera, namely *Lactococcus* (seven isolates), *Lactobacillus* (four isolates), and *Pediococcus* (one isolate). Seven *Lactococcus* isolates were identified as *L. lactis* ssp. *lactis* and *L*. *lactis* ssp. *cremoris*; four *Lactobacillus* isolates were identified as *L*. *pentosus* and *L*. *plantarum* ssp. *plantarum*; and there was only one isolate identified as *P. pentosaceus*. All isolates had the same homology ≥97% with those in the reference sequence GenBank database at NCBI (Table-2). All species isolated from two processing methods have also been isolated from various other fermented milk products such as traditional fermented dairy foods from Mongolia [17,18], China [19], Africa [20], Algeria [21], Russia [22,23] Tibet [24-26], India [27], and Iran [28].

Greater diversity of LAB species was found in dadih fermented by back-slopping method. Five LAB stains had been isolated, specifically *L. lactis* ssp. *lactis*, *L. lactis* ssp. *cremoris*, *L*. *pentosus*, *L*. *plantarum* ssp. *plantarum*, and *P. pentosaceus*. Meanwhile, at spontaneous fermented dadih, only three species were found, namely *L. lactis* ssp. *lactis, L. lactis* ssp*. cremoris*, and *L. plantarum* ssp*. plantarum* (Figure-2). Most of the identified LAB were classified in the LAB homofermentative group, apart from *L. plantarum* ssp. *plantarum* which belongs to facultative heterofermentative group. Besides LAB originating from spontaneous fermentation, there was also found heterofermentative group in back-slopping fermentation [29]. The diversity of LAB population in back-slopping fermentation is greater than spontaneous fermentation due to the addition of some products that trigger the fermentation process [15]. In the current study, the present of *L. lactis*, *Lb. plantarum*, and *L. pentosus* in dadih was assumed originated from fresh buffalo milk. In a study by Sharma *et al*. [30] and Rizqiati *et al*. [31], on isolation of autochthonous LAB in buffalo milk, *L. lactis*, *L. plantarum*, *L. pentosus*, and *L. brevis* were isolated and characterized. Meanwhile, *P. pentosaceus* suggest originated from the stable LAB community from previous dadih. The previous study indicated that *P. pentosaceus* is the most typical isolated LAB from dadih [7].

*L. lactis* contains the majority of the isolated species in both fermentation methods. *L. lactis* ssp*. lactis* and *L. lactis* ssp. *cremoris* are important group of LAB during milk fermentation and serve as starter cultures; they produce lactic acid from lactose, hydrolyze casein and also play a significant role in citric acid fermentation and flavor formation (especially *L. lactis* ssp. *cremoris*) [32-34]. When the pH value is reduced, the growth of specific microflora is triggered, namely *Lactobacillus* spp. and *Pediococcus* spp. that are both acid tolerance. The existence of these species in the final product is predominantly caused by the presence of other species who grown first and provide a suitable environment for their growth [35]. During growth, the microbes can interact and influence each other’s growth and metabolism by antagonism, metabiosis, or cell-to-cell communication [36,37].

This succession phenomenon is a process that takes place synergistically during dadih fermentation. This same phenomenon also occurs in Kivuguto, a traditional fermented milk from East Africa. LAB succession in African fermented milk mostly initiates when *Lactococcus* and *Leuconostoc* genera grown at the beginning and in the middle of fermentation (0-8 h). Isoelectric pH (4.6) will be reached by *L. lactis* after 8 h of fermentation, while *Leuconostoc* spp. need more time, approximately 14 h to reach the same pH value [38]. The acceleration of Lactobacilli genus will dominate the substrate until 24^th^ h fermentation. Acid tolerance is the property that enables that Lactobacilli to survive in the final product [39].

According to 16S rRNA gene sequence, a phylogenetic tree was constructed to describe a genetic relationship between isolates. The result (Figure-3) showed that there is no clear grouping in LAB isolates found in two fermentation methods. However, every identified group shared the same ancestor. As shown in Figure-3, *Lactobacillus* and *Pediococcus* genera were stand in same cluster, distinct from *Lactococcus* genus. Genus *Pediococcus* is an integral, not peripheral, part of the genus *Lactobacillus* [40] since the 16S rRNA gene sequence of genus *Pediococcus* was reported to fall within *Lb. casei* branch of LAB [41]. The phenotypic properties (fermentation profile in Table-3) support the genotypic characteristic of all isolates.

The diversity of LAB in the samples manufactured by the two examined methods was influenced by many factors. More extensive in-depth research is necessary to evaluate the contributing factor that affects the diversity of LAB in dadih. The different methods, recipes, raw material, environmental temperature, and location difference, may also cause some variation in strain [17]. Furthermore, anthropogenic variables (e.g., competition among sellers and organoleptic preferences) are important in shaping microbial community structure [3].

## Conclusion

The different methods in dadih fermentation significantly affected LAB diversity. The back-slopping fermentation method provided greater diversity of LAB than spontaneous fermentation in dadih (two different species had been isolated, namely *L. pentosus* and *P. pentosaceus*). Understanding the role of complex, bacterial population and diversity could be useful to adjust the fermentation methods from spontaneous to back-slopping in dadih manufacturing.

## Authors’ Contributions

MBS supervised the research. CUW and EAS carried out the analyses. MBS, DWL, IW, and EAS supervised laboratory work. CUW wrote the manuscript with the help of MBS, DWL, and IW. All authors read and approved the final manuscript.
